# Studies on the Critical Therapeutic Role of Artemisinin and its Derivatives in Melanoma: a Review of Preclinical Evidence

**DOI:** 10.1007/s11864-025-01356-y

**Published:** 2025-10-29

**Authors:** E. Liu, SiXian Bai, Ying Huang, Yaobin Pang, XueEr Zhang, Jinhao Zeng, Jing Guo

**Affiliations:** 1 Chengdu University of Traditional Chinese Medicine, Chengdu, China; 2 Dermatological Department, Hospital of Chengdu University of Traditional Chinese Medicine, Chengdu, China; 3 TCM Prevention and Treatment of Metabolic and Chronic Diseases Key Laboratory of Sichuan Province, Hospital of Chengdu University of Traditional Chinese Medicine, Chengdu, China

**Keywords:** Artemisinin, Artemisinic acid, Artesunate, Dihydroartemisinin, Melanoma, Metastatic melanoma

## Abstract

As a type of skin cancer, melanoma is characterized by a high rate of recurrence and metastasis, making it one of the leading causes of mortality associated with skin cancer. With the continuous advancement in technology, current treatment options for melanoma and metastatic melanoma have significantly improved; however, the threat posed by melanoma still warrants attention from the broader population. Artemisinin, derived from the plant Artemisia annua, is recognized as a promising drug molecule that demonstrates effective activity against both malaria and cancer. In this study, artemisinin and its derivatives (such as artemisinic acid, artesunate, and dihydroartemisinin) were shown to possess inhibitory effects on melanoma and ocular melanoma Further investigations revealed that the efficacy of these compounds is primarily linked to their ability to reduce melanin content, inhibit melanogenesis and cellular proliferation, suppress tumor growth in murine models, counteract tumor metastasis and angiogenesis, as well as promote apoptosis. The core mechanisms underlying these effects may be associated with signaling pathways such as PI3K/AKT/mTOR, MALAT918/YAP, along with those related to angiogenesis. In this study, we reviewed the inhibition of melanoma angiogenesis by natural products and its potential mechanisms using literature from PubMed, EMBASE, Web of Science, Ovid, ScienceDirect, Geenmedica, Cochrane Library and China National Knowledge Infrastructure databases. The search timeframe spans from the inception of the database to September 2025. Inclusion criteria encompass original English-language research articles, clinical trials, case reports, and relevant reviews focusing on the mechanisms, efficacy, or clinical applications of artemisinin derivatives in melanoma and ocular melanoma. Exclusion criteria include non-English literature, studies not directly related to melanoma, ocular melanoma, or the antitumour effects of artemisinin, and inaccessible Chinese-language literature. We additionally identified supplementary eligible studies through manual screening of reference lists from relevant literature.This study emphasizes the critical role of artemisinin and its derivatives in combating melanoma and ocular melanoma. The aim is to facilitate further development and utilization of these compounds while providing relevant insights for clinical research endeavors.

## Introduction

Melanoma is a tumor that evolves as a result of malignant lesions in melanocytes. It is highly malignant and most often occurs in the skin, but can also occur in different parts or tissues such as mucous membranes (including visceral mucous membranes), the uvea of the eye, and the soft meninges [1]. The main features of melanoma on dermoscopy are asymmetric structures and color inhomogeneities [2]. These include: atypical pigment networks, irregular brownish-black dots/balls/masses, pseudopods and radiolucencies (unclear borders), irregularly shaped stains/areas of hyperpigmentation, bright white streaks/linear structures, and degenerative structures [3]. Other judging criteria include blue-white streaks and pleomorphic vascular structures commonly seen in aggressive melanomas [4]. According to the 2020 ASCO Guidelines for the Melanoma describes a 3% annual increase in the incidence of melanoma in certain countries [5]. The incidence of melanoma is approximately 25 new cases per 100 000 population in Europe, 30 cases per 100 000 population in the USA, and 60 cases per 100 000 population in Australia and New Zealand [6].

From the point of view of the prevalent population, Since the 1950 s, there as been a gradual increase in the incidence of cutaneous melanoma in white populations worldwide, which has been associated with increased exposure to ultraviolet light from sunlight [7, 8]. UVA penetrates deeper into the dermis of the skin than UV B, causing skin damage and ultimately leading to skin tumors, the main mechanism of which is oxidative stress-induced DNA damage [9]while UVB causes DNA damage directly in the form of photo products, which are mainly related to the infiltration of macrophages and neutrophils [10, 11]. A family history of melanoma is a strong risk factor for the disease, and certain phenotypic traits [12]. Such as Sun sensitivity and an inability to tan, increase the risk of developing melanoma by about 50 percent [13].

Recent data reports found that the median survival of patients with melanoma was estimated at 6.2 months, with 25% of patients surviving for 1 year and 10% surviving for approximately 2 years and the median survival time for patients with metastatic melanoma is less than 1 year [14, 15].Currently, treatments for melanoma and metastatic melanoma mainly include surgery, radiation therapy, immunotherapy, targeted therapy and chemotherapy, but all of these methods have different degrees of limitations. Although surgery can effectively remove early lesions, it is difficult to cure advanced or metastatic patients, and may cause scarring or dysfunction [16]. Radiation therapy can pinpoint the tumor, but it is a long procedure, prone to skin inflammation and fatigue, and has limited effect on metastatic foci. Immunotherapy, such as PD-1/PD-L1 inhibitors, can attack tumors by activating T cells, but the response rate of Chinese patients is significantly lower than that of Western patients due to the active adenosine signaling pathway and differences in gene mutation profiles, and may cause autoimmune side effects [17]. Vemofenib-targeted therapy is 5 times more effective in patients with BRAF mutations, but resistance develops rapidly, failing after about 6 months, and the treatment is expensive [18]. Conventional chemotherapy is less selective and has significant side effects. Metastatic melanoma spreads widely, such as to the gastrointestinal tract and skin. Ipilimumab, nivolumab, and pembrolizumab, as the three main immunotherapeutic agents, have been recommended by several guidelines for standard treatment of melanoma after resection. BRIM8 and COMBI-INFECT The BRIM8 and COMBI-AD studies have also shown that patients with mutations in the BRAF V600 gene can benefit from targeted therapy. However, even when these adjuvant therapies progress, patients remain at high risk of relapse [19, 20]. In recent years, natural compounds have also been extensively studied for their antimelanoma effects, including tumor growth inhibition, induction of apoptosis, angiogenesis and metastasis inhibition, and cancer stem cell elimination [21, 22].

In addition, a considerable number of studies have reported the synergistic activity of phytochemical antimelanoma agents, as well as the enhanced effects of their synthetic derivatives and novel formulations. However, data confirming these promising effects on patients remain scarce. Artemisinin and its derivatives have been shown to produce anticancer effects by inducing cell cycle arrest, modulating signaling in apoptosis, angiogenesis and cytotoxicity to steroid receptors, and many new formulations of artemisinin are being developed in the form of carbon nanotubes, polymer-encapsulated drug particles, and so forth, which has further facilitated the exploitation of artemisinin and its compounds [23, 24]. This article highlights the anti-tumor mechanisms and potential applications of artemisinin and its derivatives in melanoma pathogenesis for ocular melanoma, with a view to facilitating preclinical studies for further development of new drugs for the benefit of melanoma patients.

## Pathogenesis of Melanoma

 Melanoma is a malignant tumor that originates from melanocytes. Melanocytes are found primarily in the skin, but are also present in the eyes, ears, central nervous system, and other areas [25]. Normally, melanocytes produce melanin in the basal layer of the skin to protect the skin from ultraviolet (UV) light. When the DNA of these cells is damaged (e.g., by prolonged exposure to UV light) and the cell’s repair mechanisms fail, because the presence of melanin makes the DNA repair machinery more sensitive to UVA-induced ROS in melanocytes than in other cell types and increases the lifetime of pyrimidine dimers in DNA, thereby increasing their risk of causing mutations [26, 27].

Currently, the most common mutations include BRAF, NRAS, and KIT. Mutated genes lead to abnormal activation of cell proliferation signaling pathways. BRAF mutations are independent of tissue origin and may provide additional opportunities for therapeutic strategies in patients with different cancers, especially those with rare cancers [28]. In melanoma or other malignant diseases, the occurrence of BRAF mutations may significantly modulate the tumor microenvironment (TME) by directly affecting tumor cells and immune cells. BRAF activation is part of the mitogen-activated protein kinase (MAPK) cell signaling pathway, and BRAF mutations can lead to unbridled activation of the downstream kinase, followed by uncontrolled cellular growth, resulting in increased cell proliferation and increased cell proliferation and survival, which provides the basis for the development of melanoma [29]. Although BRAF mutant melanoma has attracted much attention, NRAS was actually the first melanoma oncogene to be identified. In vitro studies have found that siRNA-mediated knockdown of NRAS inhibits cytokinin D1 and E2 expression [30]. Clinical studies have found that patients with NRAS-mutant melanoma tend to be older (> 55 years), have longer UV exposure, have thicker tumors at the time of diagnosis, and have a higher mitotic rate than patients with BRAF-mutant melanoma [31].Mutations in the KIT oncogene account for 3% of all melanomas. More specifically, in the skin of chronic sunburn (CSD) patients 36% exhibit melanoma of the extremities, 39% exhibit mucosal melanoma, and 28% exhibit melanoma [32, 33]. In cutaneous melanoma, the presence of SCF results in decreased expression of KIT and an increased density of methylation of the KIT promoter [34]. Additionally, it has been found that KIT can be inhibited by microRNAs (including miR-221 and miR-222), leading to a blockage of differentiation and subsequent proliferation of melanoma cells, including miR-221 and miR-222), leading to blockade of melanoma cell differentiation and subsequent proliferation, which further suggests its role in melanoma progression [35].

**Fig. 1:**
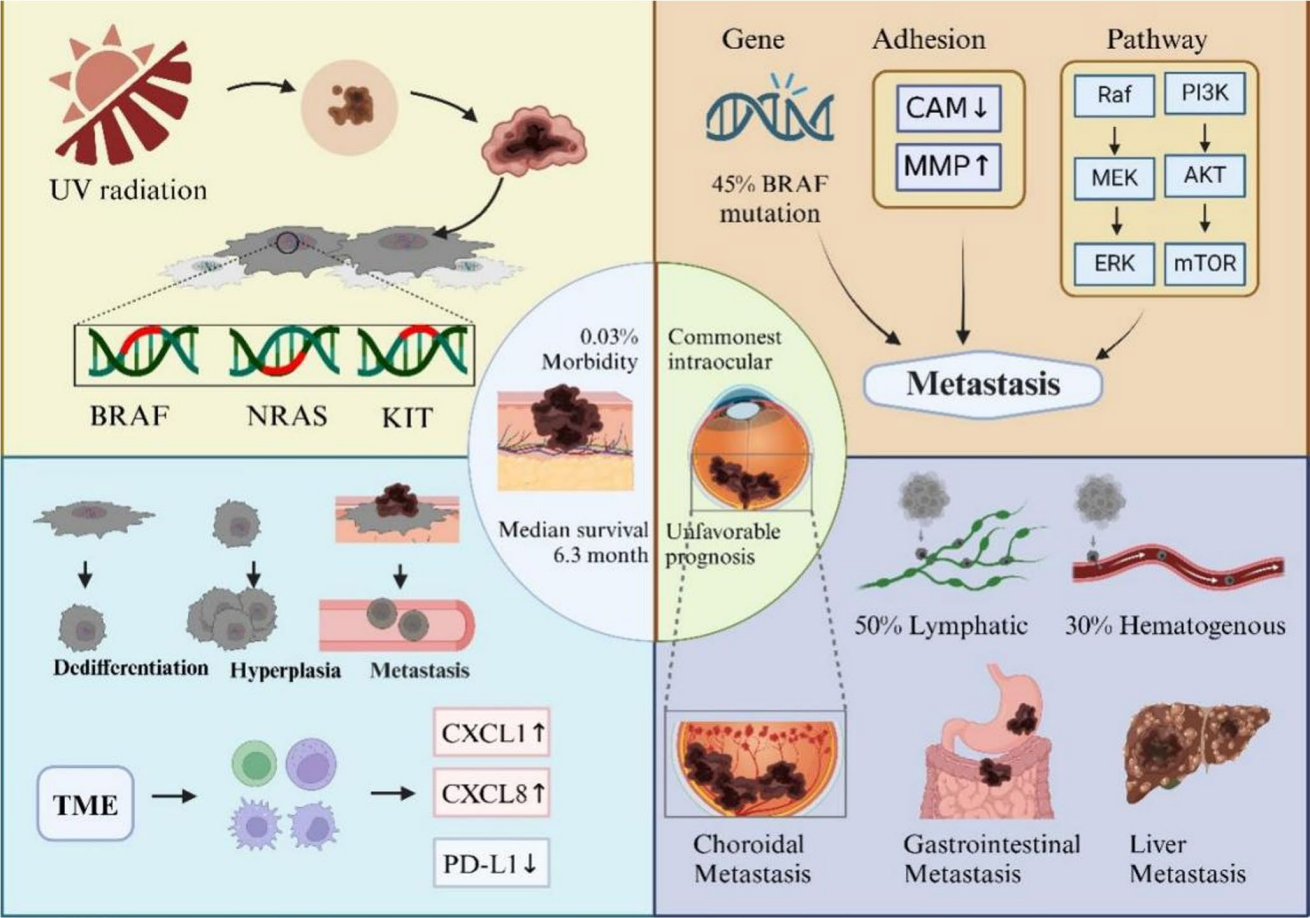
Diagram depicting the pathophysiologic mechanisms contributing to the development of melanoma and its metastasis. aThe development of melanoma is primarily associated with long-term exposure to UV light. And mutations in the BRAF, NRAS, and KIT genes and enhanced UV-stimulated expression of CXCL1 and CXCL8 are associated with the evolution of melanoma to malignant melanoma. During melanoma metastasis, the Ras/Raf/MEK/ERK and PI3K/AKT signalling pathways can influence metastasis to uveal melanoma and choroidal melanoma by affecting apoptosis and cell growth, among other key pathways

In the initial stages of the disease, Most differentiated melanocytes reside within epithelial cells, primarily the epidermis and hair follicles of the skin, but are also present in mucosal epithelium. They are also present in large numbers in the soft meninges, the inner ear, and in lower densities in many visceral organs and mutant cells may form benign nevi [2, 36]. As more and more genetic variants accumulate and transform into malignant melanoma. During this process, the cells proliferate uncontrollably, lose their normal cell polarity and organization, and begin to invade surrounding tissues and metastasize [37]. Normally, the immune system recognizes and removes the abnormal cells, preventing them from developing into a tumor. The growth and spread of melanoma is also influenced by its surrounding microenvironment, such as immune cells, fibroblasts, and vascular endothelial cells, which are able to evade the surveillance of the immune system through a variety of mechanisms that manipulate cytokines in the tumor microenvironment in order to promote tumor growth and immune escape [38]through the expression of immune-suppressing components, such as PD-L1. Clinical studies have found that hypermethylation of PD-L1 in melanoma is associated with reduced PD-L1 expression and shorter overall patient survival [39]. In addition, some cells in the tumor microenvironment, such as regulatory T cells (regulatory T cells, Tregs) and tumor-associated macrophages (TAM) in the tumor microenvironment may also help tumor cells evade immune attack by secreting immunosuppressive factors [40]. In addition, the tumor microenvironment, as a complex biological system, has a significant impact on melanoma progression. The tumor microenvironment (TME) contains many immune cells that inhibit disease progression by participating in apoptosis, production of anti-tumor cytokines, and cytotoxic responses during the first stages of melanoma carcinogenesis. NK cells bind to antigen-presenting cells (APCs) through cytokine secretion, while dendritic cells, macrophages and neutrophils are mainly responsible for phagocytosis of melanoma cells and tumor antigen presentation to activate the T-cell immune response [41].During melanoma progression, there is an increase in neutrophils in the inflammatory infiltrate of the tumor, which is mainly associated with the stimulation of CXCL1, CXCL2, CXCL3, CXCL5 and CXCL8 molecules by UV radiation [42].As a tumor progresses, melanoma cells may spread to other parts of the body through the blood or lymphatic system, resulting in metastasis [43]. This process involves multiple molecular mechanisms, including alterations in cell adhesion molecules and activation of matrix metalloproteinases (MMPs), which together promote the invasive and migratory abilities of tumor cells [44]. In vitro and in vivo studies have revealed significant expression of MMP-1, −2, −3, −9, −14, 15 and − 16 in melanoma cells, which correlates with an aggressive phenotype. In addition to destroying matrix proteins, MMP can also affect melanoma cell proliferation, adhesion, angiogenesis, survival, protease expression and migration [45]. E-calmodulin expression is observed in 10–20% of advanced melanoma lesions [46].Reduced E-cadherin expression may lead to diminished intercellular adhesion, making it easier for tumor cells to detach from the primary tumor and invade the surrounding tissue vasculature by degrading the surrounding extracellular matrix and basement membrane [47] (Fig. 1).

The Ras/Raf/MEK/ERK (MAPK) and PI3K/AKT (AKT) signaling pathways are activated by multiple mechanisms during melanoma production and metastasis. Increased activity of the MaPK pathway prevents apoptosis and induces cell-cycle progression. BRAF mutations are recognized in approximately 45% of patients with metastatic melanoma and BRAF and MEK inhibitors induce rapid response and prolong survival [48]. BRAF and MEK inhibitors induce a rapid response and prolong patient survival, the MAPK pathway is the most important pathway involved in BRAF/MEK inhibition of drug resistance, especially MAPK pathway reactivation [49]. PI3K/AKT/mTOR signaling controls cell growth through protein synthesis, and this signaling is over-activated in melanoma; targeting this pathway to reduce cancer cell growth would be helpful in the development of new drugs [50].

##  Anti-melanoma Effects of Artemisinin and its Derivatives

### Artemisinic Acid and Melanoma

#### Artemisinic Acid

Artemisinic acid (AL) is a sesquiterpene chemical compound isolated from Artemisia annua compound isolated from Artemisia annua [51]. Studies have shown that artemisinic acid can not only be used as a precursor for artemisinin synthesis [52]but also has antibacterial [53]inhibition of lipid differentiation [54]anti-tumor [55] and certain anti-malarial activity [56]. In in vivo studies in rats, artemisinic acid was highest in plasma after intravenous administration, with a clearance of 21–41 mL min^−1^ kg^−1^slower than that of the other drugs, and with a terminal half-life of about 1.5h [57].

####  Artemisinin Acid Lowers Cholesterol Levels To Reverse Melanin Synthesis by Inhibiting HMG-CoA Reductase

Recent studies have found that cholesterol is present in melanocyte membranes and. Cholesterol is important for membrane stability and is a key substrate for the synthesis of steroid hormones and vitamin D [58]. In addition, it is a major component of the lipid barrier of the human epidermal stratum corneum, and cholesterol is present in the plasma membrane of melanocytes, including melanocyte-specific organelles, melanosomes, it can increase melanin production in epidermal melanocytes [59]. There are complex interaction mechanisms between cholesterol and melanoma, involving multiple dimensions such as melanogenesis regulation, tumor cell metabolic reprogramming, immune microenvironment regulation and oxidative stress.

HMG-CoA reductase is the rate-controlling enzyme of the mevalonate lipid metabolic pathway, which plays crucial roles in oncogenesis and cancer cell stemness [60].Melanocytes synthesize cholesterol via HMG-CoA reductase and transport cholesterol via LDL/Apo-B100/LDLR, and cholesterol increases the production of melanocytes and hyperpigmentation-producing melanin in human melanoma cells (FM55) in a time- and dose-dependent manner [61].Cholesterol binds to Orai1, partially inhibiting its activity and decreasing Ca2 + ER/PM influx. Decreased Ca2 + influx leads to increased glucose uptake, which is shunted to the hexosamine pathway to increase protein synthesis, thereby promoting malignant transformation of melanocytes and increasing invasiveness [62]. At the same time, cholesterol derivatives drive tumor cell proliferation, invasion, and immune escape, inhibit CD8⁺ T-cell function and further recruit MDSCs. Currently targeted therapeutic agents such as statins inhibit cholesterol synthesis to promote cholesterol efflux. The core mechanism is that inhibition of HMG-CoA reductase lowers tissue and plasma cholesterol levels, leading to upregulation of LDLR expression in hepatic and extrahepatic tissues, which promotes clearance of plasma cholesterol-rich apolipoproteins LDL, very low-density lipoprotein (VLDL), and VLDL remnants [63]. However, statins may cause certain adverse effects such as headache, flushing, myalgia, dizziness, nausea and abdominal cramps, and rare but serious adverse effects with long-term use include rhabdomyolysis and acute hemolysis [64].

Compared to statins, artemisinin analogs are safer and can lower cholesterol levels and reverse melanin synthesis and malignant phenotypes by inhibiting HMG-CoA reductase. In vitro studies have found that incubation of human epidermal melanocytes with artemisinic acid significantly reduced melanin content in a concentration-dependent manner. The underlying reason for this is that artemisinic acid reduces cholesterol synthesis by down-regulating the HMG CoA reductase gene, which is mainly mediated by C/EBP α down-regulation [65]. This suggests that artemisinic acid may act as a hyperpigmentation inhibitor and can serve as a therapeutic agent for melanoma by inhibiting melanogenesis in human epidermal melanocytes.

### Artemisinin and Melanoma

#### Artemisinin

Artemisinin was isolated from the plant Artemisia annua in 1972 as the most promising drug molecule and its structure was analyzed by X-ray in 1979^23^. Unlike traditional Chinese medicine extraction, artemisinin needs to be extracted at low temperatures in ether, and its chemical structure will be destroyed at high temperatures. As a sesquiterpene lactone compound, the peroxy bridge in the chemical structure of artemisinin may be the main functional group for its cytotoxic effect [66]. Artemisinin has good lipid solubility, but poor water solubility and short half-life in vivo, its physicochemical and pharmacokinetic properties need to be further optimized [67]. In dermatology-related studies, artemisinin and its derivatives (artemisinin aqueous extracts, dihydroartemisinin, artesunate, etc.) have been reported to be used in the treatment of atopic dermatitis [68]eczema, psoriasis [69]rosacea [70]melanoma [71]proliferative scarring [72]skin cancer [73]photodamage of the skin [74]scleroderma [75]and allergic contact dermatitis [76]. Human studies have found that artemisinin has a Maximum concentration of 0.36 µg/ml, a peak time of 100 min, an apparent half-life of 0.62 h, a distribution half-life of 2.61 h, and a decline half-life of 4.34 hour [77].

#### Artemisinin Halt Melanoma Progression by Inhibiting Cell Growth and Metastasis

The specific role of integrin αvβ3 in tumor infiltration and metastasis is attributed to its ability to recruit and activate MMP-2 and fibrinolytic enzymes, thereby degrading stromal membranes and mesenchymal matrix components. Because integrin αvβ3 is highly expressed on the surface of a variety of tumor cells and low or absent in normal cells, it is an ideal target for tumor therapy.In melanoma, increased expression of αvβ3 integrins appears to be positively correlated with increased malignancy [78]whereas MMP, a class of structurally related zinc-dependent neutral endopeptidases, has been shown to be associated with cancer metastasis [79]and both play important roles in tumor angiogenesis, cell motility, metastasis, and tissue remodeling and repair [80]. In vitro studies found that treatment of A375M cells with 100 and 150 µM artemisinin for 72 h resulted in the best growth inhibition, with cell migration inhibition of 60% and 68%, respectively. Further, at a mechanistic level, the effect of artemisinin on the migratory ability of A375M cells May be analyzed by reducing the production of metalloproteinase 2 (MMP-2) and down-regulating the expression of αvβ3 integrins [81].

Early-stage melanoma has a high cure rate through surgical resection. Once detected late or with recurrent metastases, melanoma usually progresses faster and is insensitive to conventional radiotherapy, resulting in an extremely high mortality rate. However, almost all patients with malignant melanoma recur and metastasize after surgery and eventually die. To address this dilemma, the researchers established a post-surgical tumor model in balb/c nude mice and found that subclinical doses of artemisinin significantly blocked recurrence, metastasis, and prolonged survival in mice after tumor removal. KIT is a type III transmembrane receptor tyrosine kinase (RTK) expressing in a variety of cell types, which can regulate the downstream PI3K/AKT pathway through phosphorylation cascade [82, 83]. Artemisinin is a multi-target drug that not only regulates the c-KIT pathway, but also the complex regulatory network in cancer. The results of the study revealed his potential therapeutic efficacy in treating cancer patients after radical cancer surgery by inhibiting EMT signaling and suppressing melanoma growth [84]migration, and invasion through modulation of the KIT/PI3K/AKT pathway.

#### Artemisinin Modulates MDSC Function To Enhance Anti-PD-L1 Immunity To Treat Melanoma

Myeloid-derived suppressor cells (MDSC) are a class of bone marrow-derived, immune-suppressively active cell populations. MDSC formed in the pre-tumor immunosuppressive microenvironment are one of the major causes of resistance to immune checkpoint blockade (ICB) therapies, and inhibition of MDSC accumulation and function is critical for effective anti-PD-L1 immunotherapy [85]. A number of studies have been reported [86, 87]that an elevated percentage of MDSC in the peripheral blood of patients with advanced melanoma is associated with poorer OS and PFS as well as poor treatment outcome. Although the use of immune checkpoint inhibitors (ICIs) has significantly improved survival and response rates in patients with advanced melanoma, many patients remain resistant to ICIs. Myeloid-derived suppressor cell (MDSC)-mediated immunosuppression may be one of the main reasons for the low response rate to ICI therapy. Previous studies have reported that MDSC are enriched and activated in melanoma patients and mediate the immune escape of tumor cells by inhibiting anti-tumor T cells and NK cells, promoting tumor growth, metastasis, and decreasing the body’s therapeutic response to different melanoma therapies.In vitro studies have found that artemisinin treatment promotes T-cell tumor migration, thereby inhibiting melanoma growth in mice. In vivo studies further revealed that MDSCs lost their immunosuppressive function after artemisinin intervention and activated T-cell function by increasing iNOS expression and thereby activating T-cell function [88]the core mechanism of which was related to the activation of the PI3K/AKT, mTOR, and MAPK pathways, which promotes the reprogramming process of functional polarization of MDSCs, which may provide a new therapeutic strategy for enhancing anti-PD-L1 cancer immunotherapy.

Based on existing research, in vivo animal models provide indispensable preliminary evidence for elucidating the mechanism of action of artemisinin. Currently, xenograft mouse models are frequently employed to study melanoma disease [89]. The advantage of xenograft models lies in their use of human-derived melanoma cell cultures implanted subcutaneously into the skin or organs of immunodeficient mice, thereby mimicking the complex gaaenetic heterogeneity of cancer [90].This model is readily developed, with results obtainable within weeks. However, as most melanoma cell lines are generated under non-physiological conditions rather than within natural tissue, they no longer represent the primary tumour [91]. Furthermore, xenografts lack functional T cells, B cells, and NK cells, preventing complete simulation of potential immunomodulatory effects of artemisinin. Consequently, although mouse models strongly suggest artemisinin’s potential to significantly inhibit melanoma progression and enhance immunotherapy, these findings require further validation in humanised mouse models and clinical studies.

###  Artesunate and Melanoma

#### Artesunate

Artesunate(ART), also known as dihydroartemisinin-12-α-succinate, is a semisynthetic peroxide-bridged sesquiterpene lactone compoundderived from artemisinin [92]. ART has better absorption, solubility, and pharmacokinetics than artemisinin. Its administration can be intramuscular, oral, rectal, and intravenous [93]. ART is well known as an antimalarial drug and the mechanism of action is to cause DNA damage by producing oxidative stress and parasite mitochondrial dysfunction [94]. In addition to this, ART has a huge potential in non-malarial diseases, not only in terms of efficacy and adverse effects, but also in terms of tolerability and safety [95].When orally administered, ART has a short half-life ranging from 20 to 45 min, during which it is metabolized, through esterase-catalysed hydrolysis, to dihydroartemisinin, the active metabolite responsible for the antimalarial activity of artesunate [96]. Over the past two decades, studies have shown that ART also has a number of anticancer effects in vitro and in vivo on a variety of tumor cell lines [97].

#### Artesunate Fights Melanoma by Inducing Cell Death Signaling Pathway

Cell death is an important biological process to maintain the homeostasis of multicellular organisms. In recent years, breakthroughs in the field of cell death have been made, and following the classical apoptosis, necrosis and pyroptosis, novel modes of death such as ferroptosis and copper death have been discovered one after another [98, 99]and have been shown to have a key regulatory role in tumors [100]neurodegenerative diseases [101]and immune diseases [102].

Apoptosis is an active, genetically controlled process of programmed cell death that leads to cell destruction and cell death without involving surrounding cells or inflammatory responses [103]. Apoptosis consists of two main pathways, the intrinsic and extrinsic apoptotic pathways and ultimately leads to apoptosis [104]. It can be argued that molecular events regulating cell survival, normal growth arrest, apoptosis and cell differentiation play a key role in the overall pathogenesis of melanocyte growth [105].Artesunate had anti-proliferative and pro-apoptotic effects in melanoma A375 cells, and its inhibition of melanoma progression was associated with the expression of transcription factor-3 (STAT3) and related proteins. issue, the investigators monitored the effects of artesunate on CD4 + and CD8 + T cells, Treg and NK cells in transgenic tumor-bearing mice and non-transgenic mice, in addition to finding that artesunate inhibited melanoma cell growth and induced apoptosis, but that apoptosis of tumor cells was not attenuated with immunosuppression [106].

Ferroptosis is a newly discovered form of non-apoptotic cell death, which is significantly characterized by the accumulation of reactive oxygen species (ROS) and free iron leading to lipid peroxidation, which ultimately triggers ferroptosis [107]. ART showed significant antimelanoma effects in both in vivo and in vitro studies. In vivo studies revealed that ART directly targeted Ido1 in B16F10 cells, decreased Hic1 levels, promoted Hmox1 transcription, facilitated ROS production and lipid peroxidation, aa and induced ferroptosis in melanoma cells, leading to cell proliferation inhibition in melanoma.

#### Artesunate Inhibits Melanoma by Modulating HO-1/Fe²⁺ axis-mediated iron-dependent Oxidative Stress

Normal cells are less susceptible to receiving oxidative stress, whereas increased ROS are detected in many cancers, causing cancer cells to exhibit abnormal iron homeostasis [108]. Due to their location, melanocytes are directly exposed to environmental factors that induce oxidative stress, such as UV radiation [11]. A distinguishing feature of melanoma is the higher level of oxidative stress compared to other solid tumors [109]. This is due to the pro-oxidative state that occurs during melanin synthesis and the fact that intrinsic antioxidant defenses may be disrupted under pathological conditions.

Oxidative stress disrupts melanocyte homeostasis and leads to damage to DNA, proteins and other cellular components [110]. Altered levels of reactive oxygen species may also affect epigenetic mechanisms and promote altered gene expression, leading to melanoma development and growth. Induction of HO-1 has been shown to increase intracellular iron content [111]. To further reveal the key mechanism of HO-1 in melanoma development, in vitro studies [112] using ART on A375 melanoma cells revealed that ART selectively reduced the cell viability of melanoma cells without damaging the fibroblasts studied and induced ROS-mediated upregulation of HO-1, resulting in an increase in intracellular Fe2 + levels, further triggering the production of toxic ART free radicals, a novel finding that may open new perspectives for melanoma treatment.

### Dihydroartemisinin and Melanoma

#### Dihydroartemisinin

Dihydroartemisinin (DHA), with a molecular formula of C15H24O5 and a molecular weight of 284.35, has been demonstrated to be an effective and fast-acting antimalarial drug with low toxicity [113]. It is noteworthy that DHA is gaining attention for its low toxicity and high safety profile in exerting anticancer effects [114]. Maximum concentrations of DHA usually occur within two Hours of administration, and its mean half-life ranges from 0.49 to 3.08 h, with most half-life estimates in the 0.5–1.5 h range [115]. In the rat model, the plasma clearance of DQHS after intravenous administration was 55–64 mL min(−1) kg(−1) with a terminal half-life of 0.95 h. In contrast, the bioavailability of DQHS after intramuscular injection was 85%.A pharmacokinetic study on DHA in patients showed no differences in the concentrations of DHA within the first two days of treatment, whereas significant lower AUC of DHA was observed on the fifth day, which was implicated to be the results of physiological changes and possible auto-induction metabolism [116].

#### Dihydroartemisinin Inhibits Melanoma Progression by Regulating Mitochondria

Melanoma is the deadliest skin cancer, Alterations in mitochondrial function of melanoma cells can directly affect melanoma development and its resilience is highly dependent on mitochondrial activity and ROS signaling [117]. Mitochondrial morphology and organization and protein expression are more stable in healthy tissues, whereas in melanoma, tumor-holding and multidrug-resistant melanoma cells generate higher mitochondrial energy production, consume more oxygen, and produce more ROS [118, 119]. Studies have shown that DHA inhibits cancer cell proliferation by inducing mitochondrial apoptosis. In hepatocellular carcinoma cells, DHA acts as an upstream regulator of the Bak-mediated mitochondrial apoptotic pathway by activating Bim and NOXA [120]. In glioma cells, DHA inhibits glioma growth by targeting ERRα-mediated mitochondrial metabolism in glioma cells [121].However, whether DHA may also be involved in melanoma progression via the mitochondrial pathway does warrant further investigation.

Interestingly, in vitro and in vivo experiments have validated that DHA reduces the expression of IL-10 and IL-6 proteins in melanoma and inhibits the phosphorylation of STAT3 to induce mitochondrial apoptosis. Further studies have confirmed that DHA can counteract IL-10-dependent Treg cell inhibition to control CD8CTL function, thereby enhancing anti-tumor immunity in mice [122].In addition to mechanistic studies, how to overcome drug-induced resistance through drug delivery agents or carriers in order to reduce the toxicity of artemisinin derivatives to normal cells and enhance the toxicity to their melanoma cells has become the interest of researchers [123]. Recently, triphenylvinylpyridinium (TPVP) salts were introduced as a new class of mitochondria targeting compounds. The use of TPVP-platform to deliver anticancer drugs to mitochondria of cancer cells is an attractive strategy for cancer treatment that may also minimize normal cell toxicity, resulting in improved therapy outcome [124]. It was found that TPVP-targeted DHA showed preferential toxicity to mitochondria for cancer compared to normal cells. localization of DHA in mitochondria reduced mitochondrial function (mitochondrial membrane potential, OCR, and complex II activity), which was associated with G1-delayed, inhibition of ERK1/2 mTOR proliferation and metabolic signaling pathways [125].

It is worth noting that the anti-melanoma mechanism of ART is highly dependent on the oxidative stress it induces, and the antioxidant capacity of melanoma cells themselves is the most critical factor determining their ART sensitivity. Compared to normal melanocytes, melanoma cells typically exist in a state of persistently elevated ROS levels, resulting from their rapid proliferation, metabolic dysregulation, and activation of oncogenic signaling pathways [126]. Conversely, normal melanocytes possess a more comprehensive, modulated, and unsaturated antioxidant capacity, enabling them to effectively maintain intracellular redox homeostasis during DHA treatment without compromising viability. In other words, DHA’s pro-oxidative activity is not inherently detrimental. While ART can kill melanoma cells by inducing lethal oxidative stress, its efficacy is constrained by the complex and synergistic antioxidant defense systems within the cells [127]. Therefore, assessing a cell’s overall redox state—rather than the expression levels of individual antioxidant genes—is crucial for predicting ART efficacy and understanding its mechanisms.

#### DHA Attenuates Melanoma Progression Through an anti-oxidative Stress Pathway

Recent research suggests a causative involvement of altered redox homeostasis and reactive oxygen species (ROS)-dependent signaling in the control of metastatic melanoma survival, proliferation, and invasiveness [128–131].The sesquiterpene endoperoxide artemisinin and other semisynthetic artemisinin-derivatives including DHA constitute an important class of FDA-approved antimalarial drugs that kill plasmodium parasites through induction of iron-dependent oxidative stress [132]. In addition, DHA has shown superior anticancer effects as a pro-oxidant chemotherapeutic cancer drug in cellular, animal and clinical studies [133–135]. In vitro studies confirmed that DHA induced cell death in human A375 metastatic melanoma cells, but not in primary representative dermal melanoma cells, and that the expression of oxidative stress-responsive genes (GADD45A, GADD153, CDKN1A, PMAIP1, HMOX1, EGR1) and genotoxicity genes in DHA-exposed A375 melanoma cells (CDKN1A, PMAIP1, HMOX1, EGR1) expression was up-regulated, and in addition, moderate up-regulation of another BH3-only pro-apoptotic protein, PUMA, was detected in DHA-exposed A375 cells [136].This step suggests the potential of DHA in antimelanoma and necessitates further preclinical and clinical evaluation of antimelanoma interventions using artemisinin-derived redox antimalarials.

#### DHA Inhibits the Progression of Melanoma by Suppressing its Growth and Metastasis

The tumour doubling time (TDT) of melanoma cells can be very Long and can even occur 10 years 20 years or even more than 30 years after removal of the primary melanoma [137–139]. Long before melanoma is diagnosed, it will often metastasize to regional lymph nodes and distant sites. Among melanoma patients, metastasis to regional lymph nodes via lymphatics is the most common form of spread. Approximately 50% of metastatic patients present lymph node disease as the first site of clinically detected recurrence [140]and about 30% have no evidence of lymph node metastasis, suggesting that malignant melanoma cells metastasise only through the bloodstream [43]. However, distant organ metastases are present in approximately 30% of patients with metastatic melanoma [43].

Although DHA was found to inhibit melanoma formation, but how it affects melanoma invasion and metastasis still deserves further exploration. The research findings that Stat3 and NF-κB have a crosstalk relationship. Stat3-mediated maintenance of NF-κB activity occurs both in cancer cells and in tumor-associated hematopoietic cells [141]. IL-6, a regulator of NF-κB, can influence hepatocellular carcinoma progression by activating Stat3 [142]. Interestingly, it was found that the core mechanism of DHA’s anti-melanoma cell lung metastasis was related to the STAT3/NF-κB signaling pathway. For further in-depth studies, theresearchers used DHA to intervene in B16F10 and A375 melanoma cell lines and found that DHA significantly inhibited the proliferation and invasive ability of the above two cell lines, and significantly reduced the metastatic melanoma nodules and Ki67-positive cells in lung tissues. From a microscopic point of view, DHA promoted CD8CTL and inhibited Treg cell infiltration, promoted apoptosis and inhibited metastasis-associated proteins MMP-2 and MMP-9 in the tumor microenvironment of melanoma mice, and furthermore, the above effects were related to the STAT3/NF-κB signaling pathway [143].

Malignant melanoma, the deadliest type of skin cancer, has a high mortality rate. Current clinical regimens provide Mainly short-term effectiveness with poor Long-term outcomes. For patients with advanced Malignant melanoma, drug resistance somehow reduces their survival time by at least 5 years [144]. Hybridization and dimerization approaches have received increasing attention for their ability to reduce drug dependence and to deliver more biologically active and selective anticancer compounds. Artemisinin dimers are non-toxic to normal cells and have fewer side effects compared to monomers targeting parasites, cancer cells and viruses, and many new artemisinin-derived dimer compounds and their biological activities have recently been reported [145]. The hybrid derivatives of artesunate and coumarin were found to have low toxicity and to be able to address the problem of drug resistance that occurs during melanoma treatment, and showed significant anticancer activity against the metastatic melanoma cell line SK-MEL2 [146]. Among the five hybrids and six dimers of dihydroartemisinin and artesunate, Compared to paclitaxel (PTX) and artesunate (ARTA), hybrids 12c and 12e were active against SK-MEL3 at nanomolar concentrations, which were two orders of magnitude stronger than PTX and (ARTA). Due to their low toxicity to the healthy fibroblast cell line C3PV, these two hybrid derivatives also showed high SI values (4322 and 8813, respectively) and were able to show higher biological activity against metastatic melanoma [147].

Exosomes are nanoscale vesicles secreted by cells, exhibiting exceptional biocompatibility, low immunogenicity, the ability to traverse biological barriers, and potential for targeted drug delivery [148]. Bovine milk exosomes, owing to their wide availability, ease of extraction, and high safety profile, have emerged as highly promising drug delivery vehicles [149]. Researchers have successfully engineered an oral DHA delivery system (Exo-DHA) based on bovine milk exosomes. Findings indicate this system significantly enhances DHA’s oral bioavailability, prolongs drug retention time in vivo, and induces apoptosis while reducing migratory cells by decreasing Bcl-2 and MMP-9 expression and promoting Bax expression. This approach minimises hepatic accumulation and systemic toxicity [150]. Whilst enhancing treatment safety, this system effectively inhibits melanoma growth and lung metastasis, demonstrating markedly superior efficacy compared to free DHA. This confirms bovine milk exosomes as highly efficient and safe oral delivery vehicles, with Exo-DHA exhibiting substantial potential for clinical translation.

## Artemisinin and its Derivatives against Ocular Melanoma

### Uveal Melanoma

As far as the current reports are concerned, there are many metastatic sites of melanoma such as the ankle [151]orbital region [152]gallbladder [153]small intestine [154]larynx.and it may also involve the choroid and uvea [155, 156].

Uveal melanoma (UM) is the most common primary intraocular Malignancy in adults. UM is the second most common form of melanoma and originates primarily from melanocytes located in the uveal bundles of the eye. Being a highly aggressive disease, Although the progression of uveal melanoma is effectively controlled by radioactive plaque brachytherapy or ophthalmolysis, metastasis occurs in up to 50 per cent of patients, sometimes up to 15 years after the initial diagnosis of the disease, with a predominantly haematological metastasis pattern, and 96 per cent of the metastatic sites involve the liver [157–159].The median overall survival time for uveal melanoma is 10–13 months, and the cure rate is close to 0. Although current survival May exceed 5 years, first-line or best supportive care is effective in only 2% of cases [160]. Some immunotherapies that are effective in the treatment of cutaneous melanoma have been found to be ineffective in the treatment of UM. Conventional angiogenesis inhibitors mainly target endothelium-dependent angiogenic modes, and are ineffective in inhibiting tumor cell-coated and extracellular matrix-defined angiogenic mimetic (VM) blood supply modes [161]. The search for effective natural products will further contribute to the development of therapeutic and clinical drugs for UM.

#### Artemisinin Affects the Progression of Uveal Melanoma Through PI3K/AKT/mTOR Signaling

The PI3K (phosphatidylinositol 3-kinase)-AKT pathway is one of the most important signaling networks in cancer. It involved in cell proliferation, survival, invasion, migration, apoptosis, glucose metabolism and DNA repair [162].There is growing evidence that activation of this pathway plays a significant role in melanoma [50]. Cancer cells are partially characterised by PI3K/AKT/mTOR signalling [163]which is often overactive in cancers, including melanoma, and targeting this pathway to reduce cancer cell growth has been a major focus of drug development [50, 164].

In vitro studies have shown that artemisinin attenuated the migration, invasion and colony formation of UM cells, and promoted the loss of mitochondrial membrane potential and apoptosis of UM cells [165]. In vivo studies demonstrated that low-dose artemisinin inhibited UM melanoma tumor growth by up to 50%, whereas a 75% reduction was observed in high-dose animals by a mechanism related to PI3K/AKT/mTOR signaling, suggesting that its antitumor effects may occur by targeting this pathway [166]. A375M cells is a metastatic derivative of the parental A375P cell line displaying a medium metastatic behaviour.

#### Artesunate Combination May Affect Progression of Uveal Melanoma

Some research shows that the inhibition of growth, metastasis, and invasion of uveal melanoma by artesunate was found to be associated with a decrease in β-catenin, and its downstream targets (c-Myc, cyclin D1) [167].Verteporfin (market name Visudyne) is a benzoporphyrin derivative that has been traditionally used in the clinic for photodynamic treatment of macular degeneration [168]. Many studies have shown that Verteporfin inhibits tumor volume, growth, and Yes Associated Protein 1(YAP) expression in a wide variety of xenograft model [169]. The combination of artesunate and veniprofen enhances the apoptosis rate of human uveal melanoma C918 and inhibits the viability of human uveal melanoma C1 cells by a mechanism that may be related to the regulation of the MALAT918/YAP signaling pathway [170].

The clinical study found that the patient’s melanoma metastases resolved after a combination of formostatin and ART intervention. Another patient who received dacarbazine in combination with ART intervention experienced progressive disease stabilization followed by regression of melanoma splenic metastases and lung metastases, and this patient is still alive 47 months after the initial diagnosis of stage IV uveal melanoma. Although the number of patients treated is small, ART may be a promising adjuvant agent for the treatment of melanoma or other oncologic diseases in combination with standard chemotherapy. Its good tolerability and lack of serious side effects will facilitate the development of prospective randomized trials in the near future [171].

#### Combination therapy with dihydroartemisinin induces autophagy-mediated cell death through endoplasmic reticulum stress to treat uveal melanoma

In various human cancers, including cutaneous melanoma, autophagy-associated cell death serves as a significant immunogenic form of cell death capable of markedly enhancing tumour response to treatment. Research indicates that autophagy plays a crucial role in promoting the infiltration of dendritic cells and T lymphocytes within immunocompetent animal models [172]. Notably, elevated autophagy levels have been observed in over 40–50% of uveal melanomas, a phenomenon closely associated with tumour hypoxic microenvironments [173]. Targeted therapies addressing the autophagy pathway hold promise as novel treatment options for uveal melanoma patients who remain unresponsive to most current targeted and immunotherapeutic regimens for solid tumours.

Compared to normal melanocytes, DHA alone can inhibit UM cell proliferation but requires higher concentrations [174]. The combination of low-dose DHA with the mitochondrial uncoupler carbonylcyanide m-chlorophenylhydrazine (CCCP) significantly enhances the antitumour effect, inducing endoplasmic reticulum stress, activating the ATF4/CHOP pathway, upregulating SESN2 expression, and triggering autophagy-dependent cell death [175]. Furthermore, another decoupling agent, BAM15, also inhibits UM proliferation, indicating that the combination of mitochondrial decoupling agents with DHA represents a promising therapeutic strategy for UM. This approach is particularly suitable for local administration to enhance therapeutic efficacy and reduce toxicity.

### Choroidal Melanoma

Choroidal melanoma (CM) is the main subtype of uveal melanoma, which originates from melanocytes in the choroidal mesenchyme, and is a common intraocular malignant tumour caused mainly by malignant melanoma cells in the neural ectoderm [176].CM easily metastasises through the blood circulation, which can easily lead to blindness and death, and as Many as 50% of patients die from Tumour metastasis, of which Liver metastasis occurs in more than 2/3 of patients, with the typical characteristics of poor prognosis, high metastatic rate and high mortality [177, 178]. Radiotherapy is the most common treatment for CM, but it can lead to optic disc neovascularisation and neovascular glaucoma. Clinical studies have shown that the median survival time for patients with metastatic Choroidal melanoma is less than 1 year [179]. Factors affecting the prognosis of patients with choroidal melanoma include tumor size, age of onset, family history, and history of drug therapy, but the most critical factor is the progression of the tumor at the time of diagnosis [180]. Therefore, it is important to better understand the molecular mechanisms that control the development and progression of choroidal melanoma and to search for more effective drugs in order to develop more effective treatment strategies for patients in need.

####  Artesunate Inhibits Choroidal Melanoma Progression by Affecting Cell Growth Migration Pathways

The synergy between aberrant cell proliferation and invasive migration is the central biological basis driving the malignant progression of melanoma. In the growth dimension, melanoma cells escape growth inhibition and acquire unlimited proliferative capacity through sustained activation of pro-survival signaling pathways [181]. In the migration dimension, melanoma cells undergo epithelial-mesenchymal transition (EMT) and acquire a motile phenotype [182]. Meanwhile, overexpression of integrins (especially αvβ3) significantly enhances cell adhesion to the extracellular matrix (ECM), while degradation of the basement membrane by matrix metalloproteinases promotes cell invasion [183].Notably, there is a close crosstalk between growth and migration signaling, and this dynamic interplay allows melanoma cells to exhibit superior adaptability in local invasion and distant colonization. The synergistic modulation of this response to the growth-migration cascade by artemisinin analogs provides a unique pharmacological mechanism for the containment of melanoma metastasis.

As a member of Eph/ephrin pathway that plays vital role in tumors, EphrinA3 (EFNA3) has been proved to promote tumorigenesis in many tumors.EFNA3 has been reported to regulate Stat3 and Akt pathway signaling [184].Mechanistic studies revealed that knockdown of EFNA3 inhibited the Stat3/Akt pathway, while overexpression of EFNA3 enhanced the activation of the Stat3/Akt signaling pathway. Treatment of CM cells with ART revealed that ART inhibited EFNA3 and Stat3/Akt pathway, while overexpression of EFNA3 enhanced the growth and migration ability of ART in CM cells to a certain extent, suggesting that ART can inhibit Stat3/Akt signaling pathway by inhibiting EFNA3 to regulate CM development [185].

####  Artesunate Inhibits Choroidal Melanoma Progression by Affecting Cell Growth Migration Pathways

Neovascularisation of tumours not only provides nutrients to the tumour but also promotes metastasis of cancer cells to distant sites [186]. In melanoma, angiogenesis is a key indicator of tumour proliferation rate, survival rate and metastasis to distant organs [187].HIF-1α is one of the initiating factors in the VM process. VEGF is a key factor in the promotion of angiogenesis, and when HIF-1α is activated in tumor cells, the expression of VEGF is increased [188]. VEGF stimulates the formation of new blood vessels, which provide oxygen and nutrients, thereby supporting tumor growth and invasion. In vitro studies revealed that tumor cell proliferation was significantly inhibited and VEGF/PDGF expression was significantly downregulated after ART treatment of CM cells. Under hypoxic conditions, ART also downregulated HIF-1α/VEGF/PDGF expression. This shows that ART can effectively inhibit the ability of tumor cells to grow and form vascular mimetic structures, both under hypoxic and normoxic conditions [189]. This suggests that ART exerts an inhibitory effect on tumor growth and CM by down-regulating the HIF-1α/VEGF/PDGF signaling pathway.

The PI3K/PKB pathway, also known as the Akt/mTOR pathway, is a complex intracellular pathway that drives cell growth and tumour proliferation [190]. High expression of Ang-1 in CM promotes tumour cell proliferation, migration, and epithelial-mesenchymal transition by activating the Akt/mTOR signalling pathway, thereby driving tumour progression. Conversely, the antimalarial drug ART inhibits the Akt/mTOR pathway by downregulating Ang-1 expression, subsequently suppressing proliferation, migration, and epithelial-mesenchymal transition in CM cells. This demonstrates significant antitumour effects both in vitro and in vivo, highlighting the Ang-1/Akt/mTOR axis as a potential therapeutic target for CM [191].

## Conclusion

Bioactive compounds isolated from natural resources have been found to be most effective against tumors. Natural compounds in herbs may contain potential anti-cancer effects with minimal or no side effects [192]. Artemisinin, one of these compounds, is widely found in the Artemisia annua plant and has anticancer and antimalarial properties [23]. As a first-line antimalarial drug, the discovery of artemisinin solved the long-standing problem of chloroquine resistance and demonstrated excellent antimalarial activity [193]. However, due to the poor solubility and short half-life of artemisinin, researchers have developed artemisinin derivatives with better bioavailability [194]. Previous studies have typically discussed artemisinin’s anticancer effects as a holistic phenomenon, encompassing multiple cancer types such as lung, breast, and colon cancers. In the field of dermatology [23, 195]. Researchers found that artemisinin was superior in treating eczema and photosensitive skin conditions and the artesunate was effective in treating eczema [68, 196]. Artemisinin and its derivatives appear to be superior to commonly prescribed dermatologic drugs for the treatment of mild to moderate skin conditions.

Melanoma is a malignant tumor originating from the primitive nerve spine and can be primary in the skin, mucosa, choroid, meninges, etc. It is highly aggressive and metastatic. The incidence of melanoma is on the rise globally, and the prognosis is closely related to the clinical stage, with metastatic patients having a poor prognosis [197]. Artemisinins and their derivatives (artemisinic acid, artesunate, dihydroartemisinin) offer a unique paradigm of pharmacological intervention in melanoma therapy through a network of multi-target interactions(Table 1). Mechanistically, these compounds exhibit a triple synergistic anti-tumour effect. In the metabolic dimension, artemisinic acid regulates cholesterol biosynthesis by inhibiting HMG-CoA reductase [62]reversing the vicious cycle of melanin synthesis, while interfering with calcium signaling to arrest tumour progression; in the death-programming dimension, artesunate triggers an iron-death via HO-1/Fe²⁺ axis cascade [112]while dihydroartemisinin initiates the apoptotic pathway via mitochondrial membrane potential breakdown [136]forming a complementary cell clearance strategy; in the dimension of microenvironmental remodelling, these substances can effectively release the immunosuppressive state - not only decreasing the immunosuppressive activity of MDSC, but also promoting the CD8⁺ T cell tumour infiltration [63]and moreover blocking the IL-10/Treg immune escape axis through STAT3 signalling inhibition, providing an ideal synergistic partner for immune checkpoint inhibitors [143].

**Table 1:** Table of studies related to the key therapeutic role of Artemisinin and its derivatives in melanoma

Disease	Natural products	Models	In vivo/vitro/Clinical research	Range of dosage	Results	Reference
Melanoma	Artemisinic acid	human epidermal melanocytes	In vitro	1, 10, 50 and 100 µM	↓MITF, TRP-1,TRP-2,TRP-1mRNA, TRP-2 mRNA, c-KIT, SCF mRNA, MIF mRNA, PKA, HMG CoA	[65]
	Artemisinin	A375P in human melanoma cell lines A375M in human melanoma cell lines	In vitro	25, 100 and 150 µM	↓MMP-2, αvβ 3 inhibits the growth of melanoma cell lines	[81]
		C57BL/6 mice	In vivo	100 µM,300 µM, and 500 µM	Reduce tumor volume, tumor weight and tumor size ↑CD3 + T cell, CD8 + T cell.	
		myeloid-derived suppressor cells	In vitro	12.5,25,50,100 mg/kg	↑TNF-α, iNOS, p70 S6K mTOR ↓IL-6,IL-10,TGF-β, ARG1, PI3K/AKT, MAPK	
	Artesunate	A375 human melanoma cell line	In vitro	0.1–5µM	Decreases cell viability, reduces cell colonization, induces apoptosis, inhibits cell migration and invasion, and decreases cellular STAT3 phosphorylation. ↓STAT3, Twist, MMP-2, MMP-9	[71]
		six-week-old female C57 mice	In vivo	100 mg/kg/day	↑Hmox1,CD8 + T ↓Hic1,PD1 mRNA,	[207]
		B16F10, SK-MEL-28	In vitro	0, 10, 20, 40 µM	↑MDA, ROS, TNFα,IFNγ ↓Ki67, PD1	
		Mice (C57BL/6 background)	In vivo	1 mg/200 ml	Inhibited melanoma cell growth and induced apoptosis	[106]
		Ret-melanoma cells	In vitro	0.1–200µmol/l	↓Regulatory T cells	
		Human melanoma cell line A375 (CRL-1619)	In vitro	2.6 to 52 µM for 24–96 h.	↑ROS, HO-1,Fe2+	[112]
	Dihydroartemisinin	The B16F10 cells	In vitro	0, 5, 10, 20 mg/mL)	suppresses melanoma metastasis and proliferation ability	[122]
		8–10 weeks old female BALB/c mice	In vivo	25 mg/kg/day or 50 mg/kg/day	CD8 + CTL, IFN-γ,Bax, cleaved caspase 9, cleaved caspase 3,cleaved PARP ↓DHA GATA3, RORgt, Foxp3,IL-10,IL-6,p-state3,Bcl-2,	
		B16F10 and A375 cells	In vitro	A375: 0, 10, 20, 40 µg/ml, B16F10: 0, 5, 10, 20 µg/ml	Inhibition of proliferation and migration of melanoma cells.	[143]
		8-to-10-week-old female C57BL/6 mice	In vivo	25-50 mg/kg/day	↑CD8CTL, FasL, Cleaved-caspase-3, Cleaved-caspase-8 ↓Ki67, Treg cell, MMP-2,MMP-9, E-cadherin, Vimentin, Ezrin, N-cadherin, Snail, ZEB1, STAT3, p65	
		A375 human melanoma cells	In vitro	5 µM TPVP-DHA	↓MAPK, ERK1/2-GSK3-AKT, OCR G1-delay	[125]
		G-361, A375, and LOX human melanoma cells	In vitro	5–40 µM,48 h	Induction of A375 metastatic melanoma cell death ↑GADD45A, GADD153,CDKN1A, PMAIP1,HMOX1,EGR1, CDKN1A, PMAIP1,HMOX1,EGR1,PUMA	[136]
		C3PV, RPMI7951, PC3, MDA-MB‐231 cell lines	In vitro	0.01-1.0 µM,24 h	lower toxicity and high antimelanoma activity	[147]
		Female Sprague Dawley rats 10–12weeks old, female Swiss albino mice 8–10weeks old	In vivo	50 mg/kg DHA	Enhance the oral bioavailability of DHA, prolong the drug’s retention time in the body, reduce hepatic accumulation and systemic toxicity, and inhibit melanoma growth and lung metastasis.	[150]
		B16F10 cells	In vitro	10,20,30,40,50,60 µM,24,48,72 h	↑Bax, MMP-9 Bcl-2,survivin	
Uveal melanoma	Artemisinin	Uveal melanoma (UM-1), RGC-5, and D407 cells	In vitro	0, 3.125, 6.25, 12.5, 25, 50, and 100 µM of artemisinin for 48 h	↑caspase 3, E-cadherin, ↓ Bcl2, N-cadherin, PI3K/AKT/mTOR Attenuated the Migration, Invasion, and Colony Formation Ability of UM Cells, Inhibited Tumor Growth	[166]
	Artesunate	Primary(92.1, Mel270) and metastatic (Omm1 and Omm2.3) uveal melanoma cells	In vitro	40 µM	Reducing migration and invasion of uveal melanoma cells. ↓β-catenin, c-Myc, cyclin D1.	[167]
		The human UM cell lines C918 and M619	In vitro	0–40 μm	Artesunate significantly reduced the viability of M619 and C918 cells. caspase-3 and caspase-9 activity levels, ROS levels, and LDH release were significant in C918 cells. yAP expression and phosphorylation levels were significantly reduced, and MALAT1 overexpression significantly reduced the MALAT1/YAP signaling pathway.	[170]
		two cancer patients	Clinical research	/	Fewer side effects, longer survival	171
	DHA with carbonyl cyanide-*m*-chlorophenylhydrazone(CCCP)	Human UM cell Line 92.1	In vitro	DHA(10µM)and CCCP,2µM,	Inducing UM cell death	[175]
		Five-week male BALB/c nude mice	In vivo	DHA(1 mg/kg)and CCCP,µg/kg	↑SESN2, ATF4、CHOP, GRP78	
Choroidal melanoma	Artesunate	ARPE-19,C918,Ocm-1,Omm2.3,Mel270	In vitro	0、10、20、40、80、150 and 200µM	↓EFNA3, Stat3/Akt	[185]
		Female BALB/c nude mice aged 4 weeks	In vivo	Ip,70 mg/kg, qd	↓EFNA3	
		The human CM cell line OCM-1	In vitro	0, 1, 10, 20, 40, 80, 150, 200 µM	↓HIF-1α/VEGF/PDGF	[189]
		ARPE-19 and Ocm-1	In vitro	50 μm, 24 h	ART possesses an anti-growth activity against C918 and OCM-1 cells. ART effectively inhibits cell growth and migration by regulating Ang-1 to inhibit Akt/mTOR signaling pathwayand restrain EMT.	[191]
		BALB/c nude mice	In vivo	i.p., 70 mg/kg, qd, 14 days	ART greatly inhibited the tumorigenicity of subcutaneous xenograft model. Ang-1, p-Akt and p-mTOR	

**Fig. 2:**
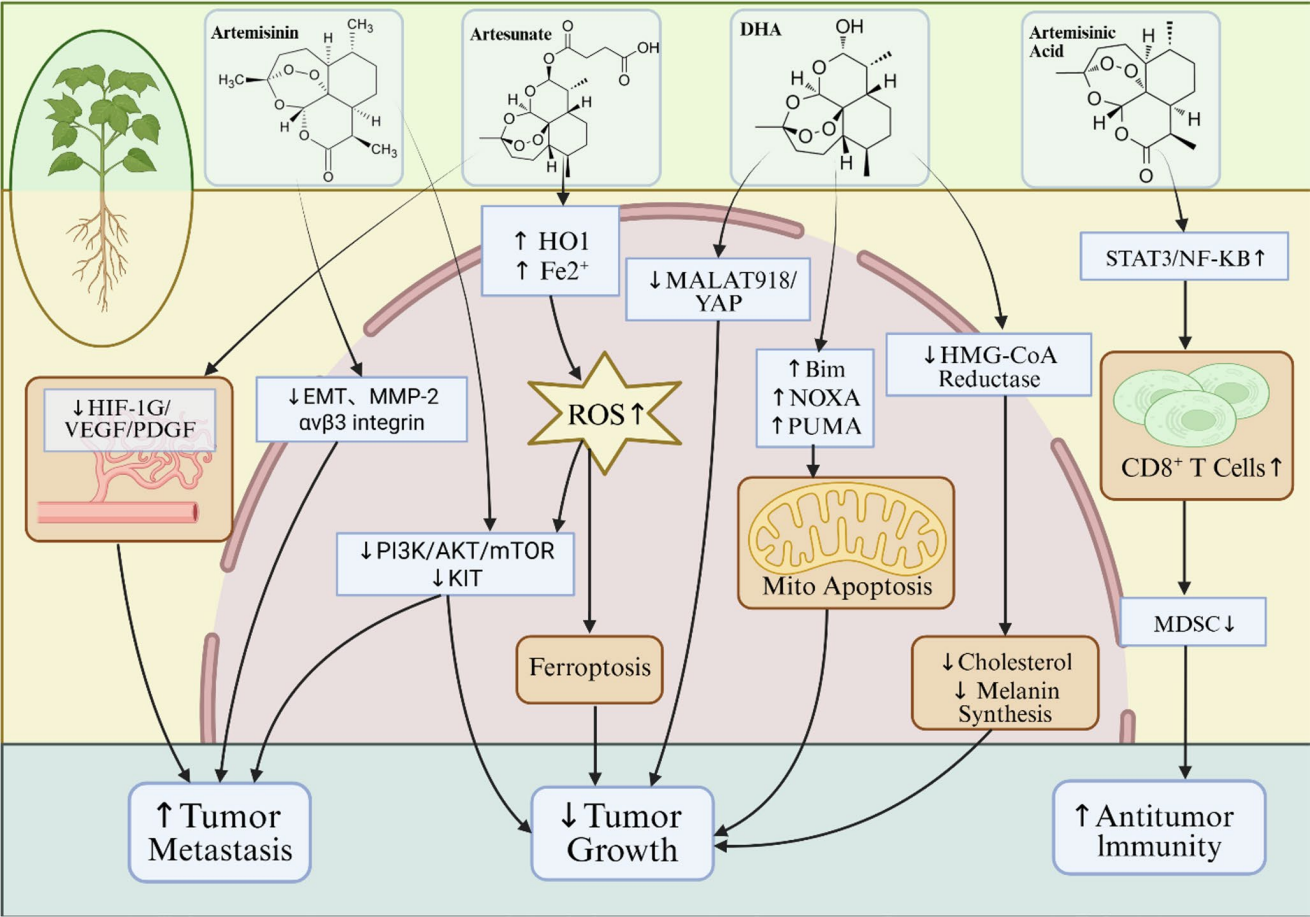
Diagram summarizing the pathways targeted by of artemisinin and its derivatives in melanoma and metastatic melanoma. Four derivatives of Artemisia annua, such as artemisinic acid, artemisinin, artesunate and dihydroartemisinin, exert their antimelanoma effects by inhibiting cell growth and metastasis, apoptosis and mitochondrial dysfunction through different pathways. The key effects of artemisinin and artesunate on choroidal melanoma and uveal melanoma are mainly related to apoptosis and angiogenesis

Metastasis is a major cause of cancer recurrence and death. This process involves complex interactions between intrinsic tumor cell properties, as well as interactions between cancer cells and multiple microenvironments [198]. Melanoma has a rapid progression and a great capacity for metastasis [199]while metastatic cutaneous melanoma is a lethal disease with a low survival rate, and most treatments can develop rapid resistance [200]. In this study, we found that artemisinin and artesunate play a key role in the progression of uveal melanoma and choroidal melanoma. In choroidal melanoma, artemisinin can target the PI3K/AKT/mTOR signaling pathway to exert tumor growth inhibition [166]while artesunate combined with venetoclax can regulate the MALAT918/YAP signaling pathway to inhibit cell viability [170]. In choroidal melanoma, the core mechanisms of artesunate tumor growth and angiogenesis, on the other hand, were related to Stat3/Akt and HIF-1α/VEGF/PDGF signaling pathways [188, 189].(Figure 2).Among the numerous signaling pathways studied for the effects of artemisinin derivatives on melanoma, the PI3K/AKT/mTOR pathway has demonstrated the most consistent and direct preclinical evidence across multiple independent studies. Artemisinin inhibits the phosphorylation of AKT and its downstream mTOR effectors, thereby suppressing cell proliferation and survival while inducing apoptosis [164]. Additionally, ART inhibits the Akt/mTOR pathway by downregulating Ang-1 expression, thereby suppressing proliferation, migration and epithelial-mesenchymal transition in CM cells [191]. This indicates consistency in PI3K/AKT/mTOR pathway data across in vitro and in vivo studies, suggesting potential for designing additional pathway-based clinical trials to advance the clinical translation and application of artemisinin derivatives. Furthermore, the STAT3/NF-κB pathway and MALAT1/miR-218/YAP pathway remain exploratory and warrant further investigation.

Melanoma, particularly advanced and metastatic melanoma, exhibits unique pathogenesis, high heterogeneity, susceptibility to drug resistance, and an immunosuppressive microenvironment [201, 202]. We systematically summarised the core regulatory mechanisms by which artemisinin and its derivatives influence melanoma cell survival and differentiation, further collating evidence of artemisinin-based drugs targeting these specific vulnerabilities in melanoma. Moreover, previous reviews on artemisinin and cancer have scarcely addressed ocular melanoma, which is a disease fundamentally distinct from cutaneous melanoma in aetiology, genetics, clinical presentation, and treatment modalities, characterised by extremely poor prognosis and a lack of effective chemotherapeutic agents. We present the first comprehensive compilation of preclinical evidence demonstrating the efficacy of artemisinin and its derivatives against uveal melanoma cell lines and animal models, aiming to provide a reference for advancing clinical research.

Artemisinin derivatives hold promising prospects as repurposed drugs in melanoma treatment due to their inherent safety profile, multiple antitumour mechanisms, and potential for combination with existing therapies. Nevertheless, their true clinical value remains to be confirmed through more rigorous clinical trials. As early as 2005, reports emerged of two patients with metastatic uveal melanoma achieving disease stabilisation or even reduction of metastatic lesions following combination therapy with artemisinin derivatives after standard chemotherapy (such as dacarbazine and formostatin) proved ineffective or led to progression [171]. These patients also demonstrated good tolerability. However, these remain isolated case reports with limited evidence strength. To date, large-scale Phase I/II clinical trials specifically evaluating artemisinin derivatives as monotherapy or in combination for melanoma remain scarce. Nevertheless, it must be recognised that substantial high-grade clinical evidence supporting its adoption as a standard treatment for melanoma is currently lacking. Future research must prioritise the design of rigorous clinical trials to clarify its precise efficacy in melanoma patients, determine optimal dosing strategies, and establish rational combination therapy regimens.

Based on existing research, artemisinin derivatives (such as ART) may show therapeutic potential when combined with certain drugs. In vitro studies indicate that the combination of ART and verteporfin significantly increases apoptosis rates in human uveal melanoma cells by regulating the MALAT1/miR-218/YAP signaling axis [170]. However, this finding requires validation through carefully designed in vivo experiments and subsequent clinical studies to assess its true therapeutic value. On the other hand, although high-level clinical evidence remains Limited, preliminary positive signals have emerged from clinical case reports. One case report described a melanoma patient who experienced regression of splenic and pulmonary metastases following combined treatment with Dacarbazine and ART, achieving survival exceeding 47 months after diagnosis of stage IV uveal melanoma [171]. Although this single-case report represents limited evidence, it provides practical rationale and motivation for further exploration of the clinical translation of artemisinin derivatives in combination with chemotherapeutic agents.

Artemisinin exhibits low water solubility, resulting in slow release and absorption when administered via intramuscular injection. Oral artemisinin tablets demonstrate poor absorption, low bioavailability, suboptimal efficacy, and a short half-life in vivo, leading to inadequate therapeutic outcomes [203, 204]. Artemisinin derivatives such as artemether share similar properties with artemisinin but also exhibit issues including rapid metabolism within the body and poor chemical stability. These characteristics make it difficult for the drug to achieve and maintain effective therapeutic concentrations at tumour sites. The clinical translation of artemisinin derivatives in melanoma treatment faces multiple obstacles, including pharmacokinetic properties, delivery technology, resistance mechanisms, and clinical evidence. To address these challenges, researchers are exploring novel delivery systems, including artemisinin nanoparticle-based drug delivery systems, self-emulsifying artemisinin formulations, novel artemisinin dosage forms, and structural modifications [205, 206]. These innovations combine traditional botanical wisdom with modern precision medicine paradigms. Only through such approaches can this ancient medicine realise its full potential in combating melanoma, achieving a qualitative leap from laboratory discovery to clinical treatment concepts. Future research will further propel these promising compounds from the laboratory to the clinical setting, ultimately benefiting patients.

As modern technology continues to evolve, in order to break through the current translational bottlenecks, future efforts could focus on exploring the combination of artemisinin compounds with the field of materials science to delay melanoma progression, and given the increasingly interdisciplinary nature of the field, the potential value of combining artemisinins and their derivatives with nanomaterials, hydrogels, and other matrices to enhance melanoma inhibition continues to warrant further investigation. In addition, combination programmes with synergistic properties of artemisinin-based compounds can be designed. For example, artemisinin compounds could be combined with ferroptosis inducers, synergised with YAP inhibitors, or paired with mitochondria-mediated pathways to inhibit melanoma progression. In order to further promote the development of clinical research and the translation of scientific and technological achievements, and to improve the efficacy of artemisinin-based compounds in the treatment of melanoma, the optimisation of their extraction techniques and the standardisation of their clinical dosage are essential to increase their utilisation and to facilitate the development of new clinical drugs, which will be the focus of future research.

## Key References

- Long, G. V., Swetter, S. M., Menzies, A. M., Gershenwald, J. E., and Scolyer, R. A., Cutaneous melanoma. The Lancet, 2023, 402, 485–502.
  - This reference is of outstanding importance because of its systematic coverage of disease definition, pathogenesis, clinical presentation and diagnosis, and tumour thickness-based staging and management. The paper also provides authoritative global epidemiological data that provide insight into the long-term trend of increasing incidence.
- Zhou, Z., Farhan, M., Xing, X., et al., Artemisinin Suppressed Melanoma Recurrence and Metastasis after Radical Surgery through the KIT/PI3K/AKT Pathway. Int J Biol Sci, 2025, 21, 75–94.
  - This reference is of outstanding importance because of this study confirms that artemisinin significantly blocks tumour recurrence and metastasis and prolongs survival. The breakthrough lies in revealing the molecular mechanism by which artemisinin exerts anti-tumour effects through inhibiting the c-KIT/PI3K/AKT signalling pathway, which provides a theoretical basis for therapeutic efficacy.
- Liu, W.-S., Chen, Z., Lu, Z.-M., et al., Multifunctional hydrogels based on photothermal therapy: A prospective platform for the postoperative management of melanoma. *J Control Release*, 2024, **371**, 406–428.
  - This reference is of importance because it provides directions for future research and development of multifunctional biomaterials through an interdisciplinary strategy that investigates postoperative oncology management and exploits the superiority of hydrogel technology.

## Data Availability

The datasets used or analysed during the current study are available from the corresponding author on reasonable request and all figures and forms are original.
